# Amyloid-like aggregates of neuronal tau induced by formaldehyde promote apoptosis of neuronal cells

**DOI:** 10.1186/1471-2202-8-9

**Published:** 2007-01-23

**Authors:** Chun Lai Nie, Xing Sheng Wang, Ying Liu, Sarah Perrett, Rong Qiao He

**Affiliations:** 1 State Key Laboratory of Brain and Cognitive Science, Institute of Biophysics, 15 Datun Rd, Chaoyang District, Beijing 100101, China; 2 National Laboratory of Biomacromolecules, Institute of Biophysics, 15 Datun Rd, Chaoyang District, Beijing 100101, China; 3 Graduate School, Chinese Academy of Sciences, 19 Yuquan Rd, Shijingshan District, Beijing 100049, China

## Abstract

**Background:**

The microtubule associated protein tau is the principle component of neurofibrillar tangles, which are a characteristic marker in the pathology of Alzheimer's disease; similar lesions are also observed after chronic alcohol abuse. Formaldehyde is a common environmental contaminant and also a metabolite of methanol. Although many studies have been done on methanol and formaldehyde intoxication, none of these address the contribution of protein misfolding to the pathological mechanism, in particular the effect of formaldehyde on protein conformation and polymerization.

**Results:**

We found that unlike the typical globular protein BSA, the natively-unfolded structure of human neuronal tau was induced to misfold and aggregate in the presence of ~0.01% formaldehyde, leading to formation of amyloid-like deposits that appeared as densely staining granules by electron microscopy and atomic force microscopy, and bound the amyloid-specific dyes thioflavin T and Congo Red. The amyloid-like aggregates of tau were found to induce apoptosis in the neurotypic cell line SH-SY5Y and in rat hippocampal cells, as observed by Hoechst 33258 staining, assay of caspase-3 activity, and flow cytometry using Annexin V and Propidium Iodide staining. Further experiments showed that Congo Red specifically attenuated the caspase-3 activity induced by amyloid-like deposits of tau.

**Conclusion:**

The results suggest that low concentrations of formaldehyde can induce human tau protein to form neurotoxic aggregates, which could play a role in the induction of tauopathies.

## Background

Although many studies have been done on methanol and formaldehyde intoxication [1,2], none of these address the contribution of protein misfolding to the pathological mechanism, in particular the effect of formaldehyde on protein conformation and polymerization. Damage of neuronal cells caused by misfolded protein aggregates is a subject of intense research interest. It has become increasingly clear that many neurodegenerative diseases are related to aggregation and deposition of misfolded proteins, such as tau [3-5], beta amyloid [6-9], alpha-synuclein [10,11] and polyglutamine aggregates [12,13]. The abnormal deposition of misfolded protein causes the malfunction of a distinctive set of neurons [14]. Alzheimer's disease and some other dementias are related to pathological deposition of proteins. Tau is a microtubule-associated protein, which is the main constituent of paired helical filaments (PHFs) present in neurofibrillary tangles [4,5]. In neurodegeneration, tau protein accumulates in lesions composed of fibrillar aggregates displaying the cross β-sheet diffraction pattern of "amyloid" [15]. Interestingly, neurofibrillary tangles have been found in brains of chronic alcoholics possessing neuropathological signs of thiamine-deficiency, suggesting that tau misfolding may be involved in the alcohol-induced pathological pathway [16-18].

Methanol ingestion is an important public health concern because of the selective actions of its toxic metabolites, formaldehyde and formic acid, on the retina, optic nerve and central nervous system [1]. Severe and even fatal illness has been reported after illicit consumption of "industrial methylated spirits" [2]. Methanol is oxidized by alcohol dehydrogenase to produce formaldehyde, which is further oxidized to formic acid by formaldehyde dehydrogenase. Metabolism of methanol to formaldehyde via peroxisomal enzymes has been demonstrated in rat retina in vitro [19], and the presence of cytoplasmic aldehyde dehydrogenase activity has been demonstrated in several regions of the rat and mouse eye, including the retina [20,21]. Susceptibility to methanol toxicity is dependent upon the relative rate of formate clearance. However, methanol toxicosis induces progressive damage to the central nervous system. It is hard to explain this chronic damage by local accumulation of formic acid alone.

Formaldehyde is a common environmental contaminant found in paint, clothes, medicinal and industrial products, and is a component of diesel and gasoline exhaust [22,23]. Recently, Sarsilmaz and colleagues have reported that formaldehyde exposure may cause various morphological changes in the rat brain [24,25]. Neurotoxic effects have been also confirmed by acute and subacute formaldehyde exposure in mice [26]. Pitten *et al*. have classified formaldehyde as "probably neurotoxic" [27], because they found rats exposed to formaldehyde need more time and make more mistakes than the animals of the control group while going through a maze. As a crosslinking agent, formaldehyde readily reacts with thiol and amino groups [28], causing polymerization of proteins. In semicarbazide-sensitive amine oxidase (SSAO)-mediate pathogenesis of Alzheimer's disease, formaldehyde interacts with β-amyloid and produces irreversibly cross-linked neurotoxic amyloid-like complexes [29-31]. Therefore, the potential effect of formaldehyde on protein misfolding may be significant, even if formaldehyde remains in the human body for only a short time.

Here, we examine the role of formaldehyde in induction of protein misfolding. In particular, we investigate the effect of formaldehyde on the aggregation of human neuronal tau in vitro and the toxicity of tau aggregates in mammalian neuronal cells. The results imply that low concentrations of formaldehyde are sufficient to induce formation of amyloid-like tau aggregates, which in turn induce apoptosis of both human neuroblastoma cells (SH-SY5Y) and rat hippocampal cells.

## Results

### Formaldehyde at low concentrations induces tau to form amyloid-like aggregates

**Figure 1:**
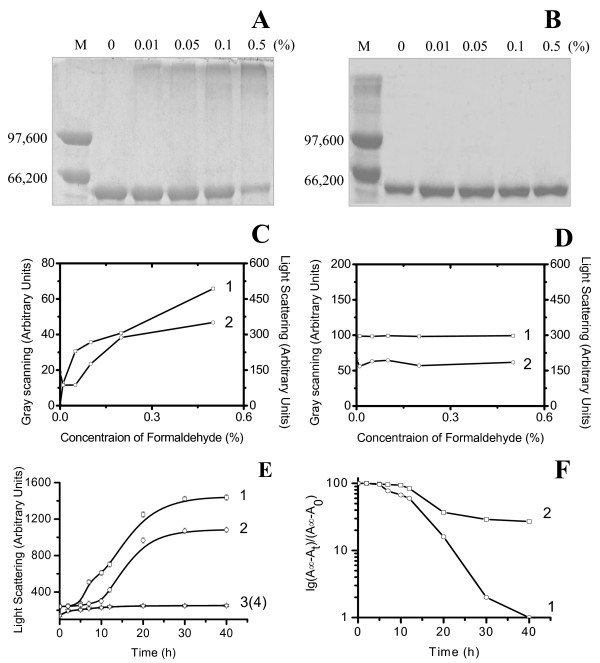
**Effect of formaldehyde on tau aggregation at different concentrations**. (A) Recombinant htau-40 (20 μM final concentration) was incubated with formaldehyde at desired concentrations in 100 mM phosphate buffer (pH 7.2) at 37°C for 24 h and aliquots (10 μl) were loaded for 10% SDS-PAGE. Lane M contains molecular mass standards. (B) BSA was used as a control. (C) Gray densities of tau polymers on SDS-PAGE were measured (curve 1) and changes in the light scattering of tau with formaldehyde at different concentrations were detected (curve 2). (D) The gray densities of BSA monomers from panel B (curve 1) and the light scattering of BSA (curve 2). (E) Tau-40 (1.2 μM final concentration) was incubated with 0.1 % formaldehyde and changes in the light scattering at 480 nm were measured at different time intervals in the presence (curve 1) or absence (curve 2) of formaldehyde. BSA alone (curve 3) or BSA incubated with formaldehyde (curve 4) was used as controls. (F) The same data as shown in panel E is plotted on a semi-logarithmic scale [36].

In order to investigate the potential of formaldehyde to induce protein misfolding leading to neurodegenerative disease, we studied the effect of low concentrations of formaldehyde on the biochemical and biophysical properties of human neuronal tau. Incubation with increasing concentrations of formaldehyde from 0.01–0.5% was observed to result in formation of increasing amounts of SDS-insoluble aggregates of tau, as detected by SDS-PAGE (Fig. 1A; Fig. 1C, curve 1) or light scattering (Fig. 1C, curve 2), similar to our previous study [32,33]. In contrast, when BSA was incubated with formaldehyde under the same range of conditions, no significant degree of aggregation could be detected by SDS-PAGE (Fig. 1B; Fig. 1D, curve 1) or by light scattering (Fig. 1D, curve 2). This suggests that tau is more susceptible to the effects of formaldehyde than typical globular proteins.

We then compared the time course of the aggregation reaction in the presence (Fig. 1E, curve 1) and absence (Fig. 1E, curve 2) of 0.1% formaldehyde by monitoring the degree of light scattering. In both cases a sigmoidal curve was observed. However, the presence of formaldehyde both reduced the lag time for aggregation and resulted in a greater degree of light scattering (Fig. 1F). The presence of formic acid had no detectible effect on tau aggregation (data not shown). This further suggests that formaldehyde promotes aggregation of tau. In contrast, incubation of BSA under the same conditions showed little aggregation in the absence (Fig. 1E, curve 3) or presence (Fig. 1E, curve 4) of formaldehyde.

**Figure 2:**
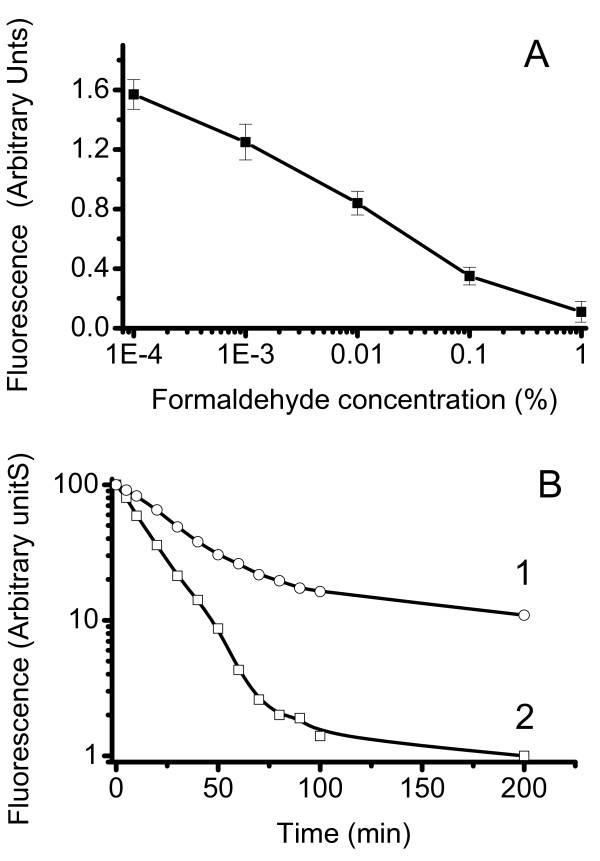
**Reaction of formaldehyde with amino groups of neuronal tau**. (A) Neuronal tau (final concentration 0.1 μM) was resuspended in phosphate buffer containing OPT (20 times molar excess compared to protein) in the presence of formaldehyde at different concentrations at 37°C for 120 min. The fluorescence (Ex340 nm/Em455 nm) was then measured. (B) Under the same conditions, tau was incubated with (curve 1) or without (curve 2) 0.005% formaldehyde and 2 μM OPT. Aliquots were then taken to measure the fluorescence at different time intervals. The data were plotted on a semilogarithmic scale as described by Tsou et al. [59].

OPT is commonly used as a fluorescent probe to detect both α- and ε-amino groups of a protein [34]. As shown in Fig. 2A, the fluorescent intensity at 455 nm decreased as the formaldehyde concentration increased. In 1% formaldehyde solution, the fluorescence could hardly be detected under the same conditions, indicating that formaldehyde competed with OPT in the reaction with the amino groups. The time course of the fluorescence changes in the presence and absence of 0.005% formaldehyde monitored using OPT showed a marked difference (Fig. 2B). The first order rate of the fluorescence change in the absence of formaldehyde (~1.03 × 10^-3^s^-1^) was greater than that in the presence of formaldehyde (~3.66 × 10^-4^s^-1^). This demonstrates that formaldehyde reacts with protein tau and blocks amino groups.

**Figure 3:**
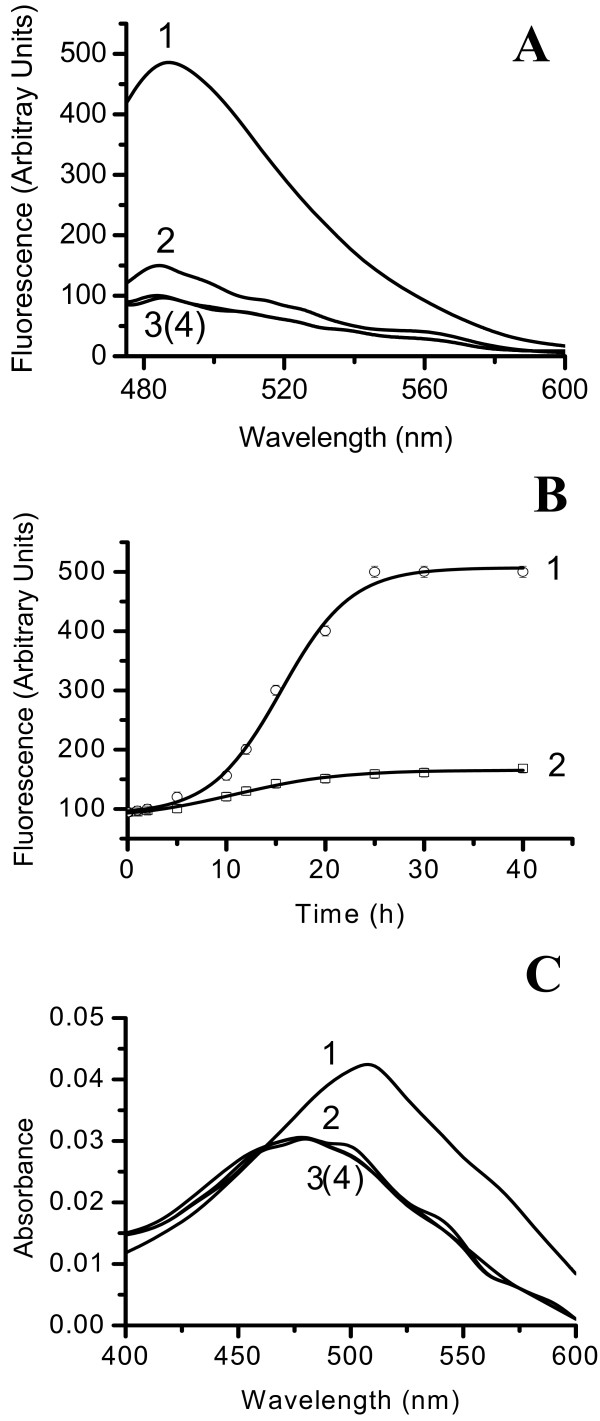
**Changes in fluorescence spectra of thioflavin T and absorption spectra of Congo Red in the presence of tau deposits**. (A) Thioflavin T (10 μM final concentration) was incubated with 0.1% aldehyde-treated tau (2 μM) in 100 mM potassium phosphate buffer pH 7.2 for 15 min before measurement. Emission spectra of ThT were recorded (excitation at 450 nm) in the presence of formaldehyde-treated tau (curve 1), self-aggregated tau (curve 2), acetaldehyde-treated tau (curve 3) and in the absence of protein (curve 4). (B) Kinetics of the increase in the fluorescence emission of ThT incubated with formaldehyde-treated tau (curve 1) or self-aggregated tau (curve 2). (C) Under the same conditions, Congo Red (5 μM final concentration) was incubated with formaldehyde-treated tau (curve 1), acetaldehyde-treated tau (curve 2) or native tau (curve 3) for 15 min before measurement. Congo Red alone is shown as a control (curve 4).

**Figure 4:**
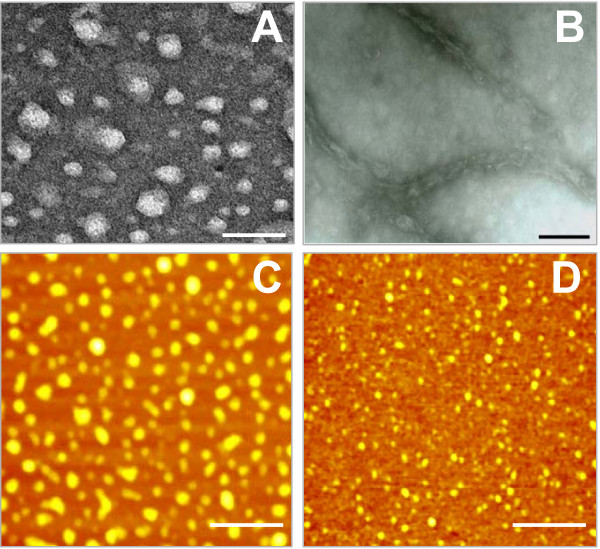
**Tau deposits were imaged by transmission electron microscopy and atomic force microscopy**. Tau deposits were formed by incubation of tau with formaldehyde in 100 mM sodium phosphate, pH 7.2, for one day. (A) Formaldehyde-treated tau deposits stained with uranyl acetate were observed under the electron microscope (Bar: 100 nm); (B) Tau (40 μM) was incubated with heparin (1 mg/ml) under the same conditions (Bar: 50 nm). (C) Formaldehyde-treated tau deposits observed by AFM (Bar: 125 nm). (D) Tau alone as a control was observed by AFM (Bar: 125 nm).

The time course of tau aggregation in the presence and absence of formaldehyde was also monitored using the fluorescent dye ThT, which was considered to be highly specific for amyloid-like structure [35]. Under the conditions used, self-aggregated tau showed only minor change in the ThT fluorescence (curve 2, Fig. 3A and 3B). Acetaldehyde-treated tau likewise did not show any significant change in the ThT spectrum (curve 3, Fig. 3A), compared with a protein-free control (curve 4, Fig. 3A). Interestingly, however, formaldehyde-induced aggregation of tau showed a significant change in the intensity of ThT fluorescence (curve 1, Fig. 3A), and the sigmoidal time course of aggregation detected by ThT binding (curve 1, Fig. 3B) was similar to that observed by light scattering (curve 1, Fig. 1E). Formaldehyde-treated tau was also found to bind Congo Red, causing a red shift in the spectral maximum from 470 nm to 510 nm (curve 1, Fig. 3C), as observed for amyloidogenic tau peptides [36], compared with acetaldehyde-treated tau, native tau or Congo Red alone as controls (curves 2, 3 and 4, Fig. 3C). Negatively-stained electron micrograph images of the formaldehyde-induced aggregates showed dense granules (Fig. 4A), whereas self-aggregated tau as control showed PHF-like structures by transmission electron microscopy (Fig. 4B), similar to the results reported previously [5]. By AFM, protein tau treated with 0.05% formaldehyde also showed globular particles on the mica surface. The horizontal diameter of the tau particles was 18.65 ± 2.66 nm (mean ± SD, Fig. 4C) about twice the size of native tau (9.94 ± 1.96 nm, Fig. 4D). However, fibril-like aggregates could not be observed in the formaldehyde-treated tau that was incubated for over a week.

Consequentially, we measured the activity of the different types of tau aggregates in tubulin assembly. While the spontaneously formed aggregates and those formed in the presence of acetaldehyde, maintained a relatively high level of residual activity, the formaldehyde-induced aggregates of tau were practically inactive in promoting tubulin assembly (Table 1). Together, these results indicate that the presence of formaldehyde promotes the formation of amyloid-like aggregates of tau, causing tau to become inactive in tubulin assembly.

### Conformational changes of amyloid-like tau aggregates

**Figure 5:**
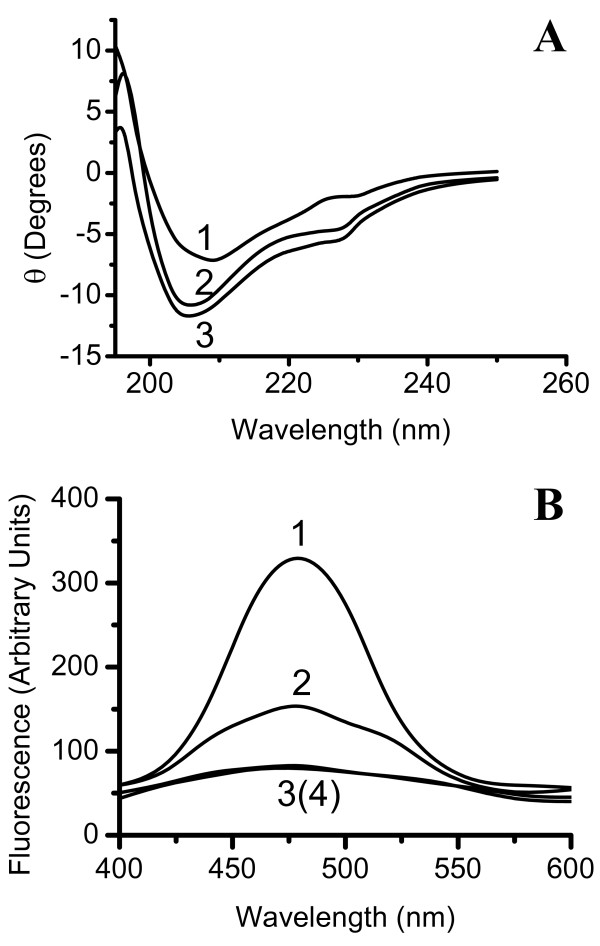
**Conformational changes of polymerized tau in formaldehyde solution**. (A) Circular dichroism spectra of 2 μM native tau (curve 2), and tau incubated with 0.1% formaldehyde (curve 1) or acetaldehyde (curve 3) at 37°C for 24 h. Scattering contributions of the aldehyde were subtracted from the spectra. (B) Changes in the fluorescence of tau in the presence of ANS. Tau (1.2 μM final concentration) was incubated in 100 mM phosphate buffer (pH 7.2) with or without 0.1 % aldehyde at 37°C overnight and then ANS (molar ratio: tau/ANS = 1/40) was added. Changes in the ANS fluorescence spectra at 480 nm for tau incubated with formaldehyde (curve 1), acetaldehyde (curve 3) or without aldehyde (self-aggregation, curve 2) were measured by excitation at 350 nm. Formaldehyde alone (curve 4) is shown as a control.

The ability of tau aggregates to bind the dyes ThT and Congo Red suggests that the aggregated tau contains relatively more β-sheet structure compared with the natively unfolded protein. To prove this prediction, htau 40 was incubated in the presence or absence of formaldehyde and then examined by circular dichroism (CD) spectroscopy. In the absence of formaldehyde, both native tau and acetaldehyde-treated tau showed CD spectra typical for an unfolded protein as reported [37,38], with a broad minimum of ellipticity centered at 205 nm (curves 2 and 3 respectively, Fig. 5A). The presence of formaldehyde led to a noticeable change in the spectra so that the minimum became wider and was shifted toward higher wavelength, suggesting a substantial change from random coil to β structure consistent with the premolten globule folding state (curve 1, Fig. 5A), similar to previous reports of an increase in β conformation in tau aggregation [37-39]. These data further support that formaldehyde-induced tau aggregates contain partially folded structure that is enriched in β-sheet content in contrast to the initial conformation.

In addition to its secondary structure signature, the premolten globule state is characterized by a partially collapsed structure with a loosely packed hydrophobic core. To confirm that the aggregates had partially folded character, htau40 was incubated with formaldehyde and then examined for the ability to bind ANS, a fluorescent probe of surface-exposed hydrophobic patches [40]. ANS in buffer alone (not shown) fluoresced weakly at the optimum wavelength (Ex: 350 nm; Em: 480 nm) [39], which was unaffected by addition of increasing concentrations of formaldehyde in the absence of tau (curve 4, Fig. 5B). In contrast, ANS in the presence of formaldehyde-aggregated tau fluoresced brightly (curve 1, Fig. 5B). A slight increase in fluorescence intensity was detected for self-aggregated tau (curve 2, Fig. 5B), indicating some PHF-like structures could form as described previously [5,32]. Note that no marked increase in fluorescence intensity was detected for acetaldehyde-treated tau (curve 3, Fig. 5B). This suggests that the enhanced fluorescent signal observed came from the binding of ANS to altered conformations of tau induced by formaldehyde.

### Amyloid-like tau promotes apoptosis of neuronal cells

**Figure 6:**
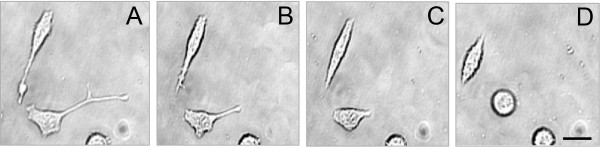
**Contrast microscope image of cells treated with amyloid-like tau**. The same SH-SY5Y cells were imaged after incubated with tau deposits (2 μM) for 0, 24, 48 or 72 h (A-D). Cells were visualized by inverted contrast microscopy. Bar = 25 μm.

**Figure 7:**
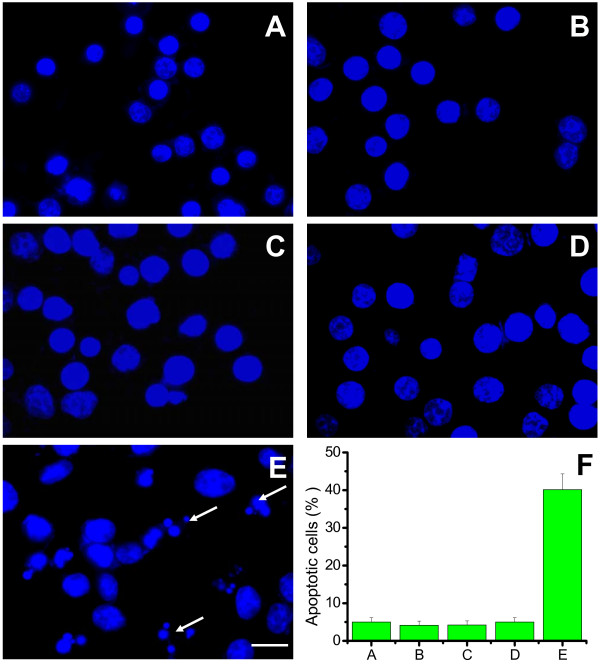
**Hoechst staining of cells in the presence of amyloid-like tau**. Treatment with formaldehyde-treated tau resulted in apoptotic death. Note that formaldehyde was removed from the protein samples by ultrafiltration. SH-SY5Y cells were treated for 3 days with tau deposits (2 μM) induced by pretreatment with 0.1% formaldehyde. The cells were collected and stained with Hoechst 33258. Nuclei were visualized by fluorescence microscopy. Bar: 25 μm. (A) Control culture in DMEM without serum. (B) Cells treated with self-aggregated tau. (C) Cells treated with 0.1 %-acetaldehyde-treated tau. (D) Cells treated for 72 h with the mock solution without formaldehyde were collected and stained with Hoechst 33258. (E) Cells treated with 0.1 %-formaldehyde-treated tau. The arrows designate the presence of apoptotic nuclear profiles. Data are expressed as a percentage of the control (cells treated with vehicle alone) and presented as the mean ± SEM (n = 6). (F) Statistics of apoptotic cells from A-E.

In order to investigate whether amyloid-like tau has an effect on neurons, amyloid-like tau was added to neurotypic SH-SY5Y cells after the residual formaldehyde had been removed completely from the protein-deposits by ultrafiltration (see Methods). Figure 6 illustrates that amyloid-like tau induces marked axonal atrophy and finally the cells shrink into a spherical shape in the neuroblastoma culture. To tell whether this deposit-induced cell death represented apoptosis, we examined two features of apoptosis: the morphology (nuclear condensation and fragmentation) and the biochemical (caspase activity) changes of the cells. As shown in Fig. 7, morphological evaluation of neuronal cultures using Hoechst 33258 staining with fluorescence microscopy revealed a significant increase in the number of cells showing nuclear condensation and fragmentation after incubation with amyloid-like tau for 3 days (Fig. 7E). Under these conditions, 0.1% formaldehyde solution ultrafiltered with 100 mM phosphate buffer (pH 7.2) was used as a control. No marked characteristics of apoptosis could be detected by Hoechst 33258 (Fig. 7D) in the presence of the solution whose formaldehyde was removed by the ultrafiltration. Further, no effect on cell morphology could be detected in the presence of a similar dose of self-aggregated tau (Fig. 7B) or acetaldehyde-induced tau (Fig. 7C).

**Figure 8:**
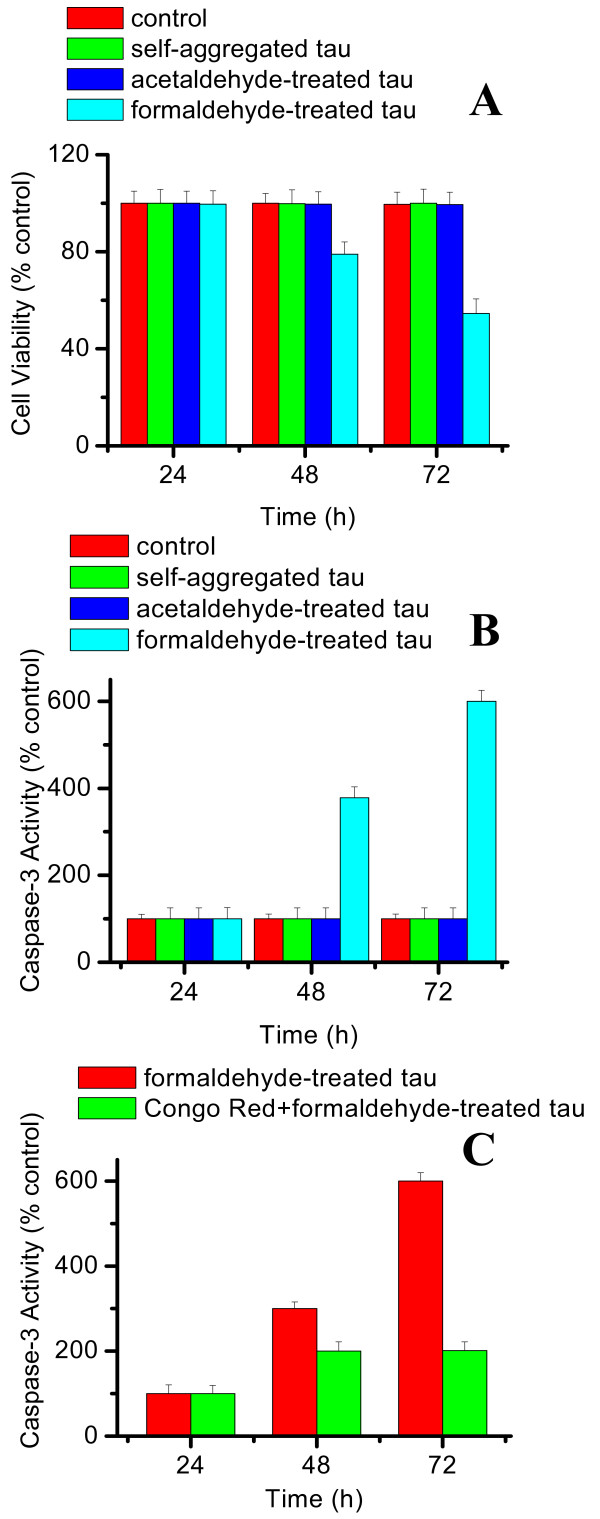
**Cell viability and Caspase-3 activity measurements over time**. (A) Cell viability was measured by the MTT assay as described in Materials and methods. (B) After SY5Y cells were treated with different tau samples (2 μM) for 24 h, cell lysates were collected at the times indicated and used to measure caspase-3 activity. (C) SH-SY5Y cells were treated with tau deposits (2 μM) in the presence or absence of 10 μM Congo red. Cell lysates were collected at the times indicated and caspase-3 activity was measured. Data are expressed as a percent of the control (cells treated with vehicle alone) and presented as the mean ± SEM (n = 6).

The effect of amyloid-like tau on cell viability was estimated using the MTT assay [41]. As shown in Fig. 8A, the percentage of viable cells in the culture decreased significantly over the course of 72 h after treatment with amyloid-like tau. In contrast, the presence of self-aggregated tau or acetaldehyde-treated tau had no effect on the number of viable cells under the same conditions. Over the same time period, the caspase-3 activity of cytosolic protein extracts from the SH-SY5Y cells treated with amyloid-like tau was observed to increase, while the caspase-3 activity for the control cultures remaining constant (Fig. 8B). The effects of Congo Red on the ability of formaldehyde-aggregated tau to induce apoptosis in the SH-SY5Y cell-culture were examined. The caspase-3 activity produced in response to treatment with formaldehyde-aggregated tau was reduced significantly by addition of Congo Red (Fig. 8C). Congo Red alone caused no detectable effect on cell viability (not shown). This then suggests that the ability of Congo Red to bind to amyloid-like tau confers a protective effect on neurons, as has been observed in the case of β-amyloid [42].

**Figure 9:**
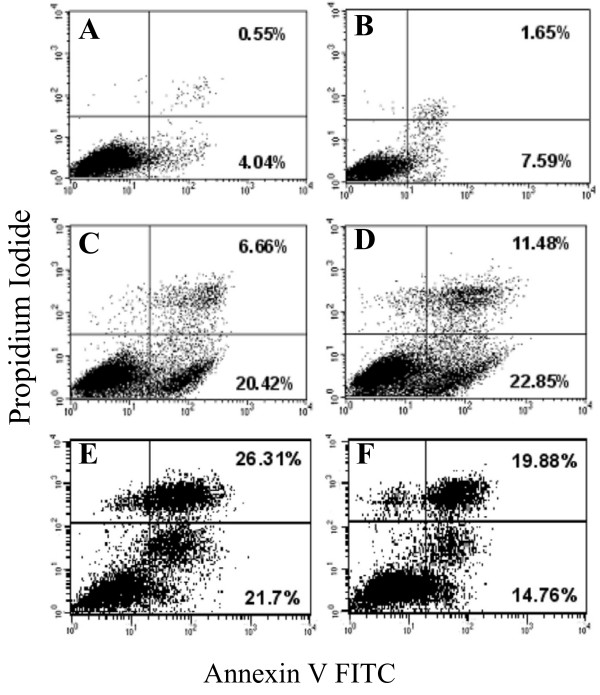
**Amyloid-tau treatment induces rat hippocampal cell apoptosis**. Flow cytometric analysis of primary hippocampal cells after treatment with 2 μM formaldehyde-treated tau. The percentage of apoptotic cells were characterized as those that stained with Annexin-V and excluded PI (see Materials and Methods). (A) Control cultured in DMEM without serum. (B), (C) and (D) represent the results of cells exposed to 0.1%-formaldehyde treated amyloid-tau for 24, 48, and 72 h, respectively. (E) and (F) represent cells incubated for 72 h with 0.05%- or 0.01%- formaldehyde-treated tau, respectively. Data represent the mean values of three independent experiments.

In order to further investigate the neurotoxicity of amyloid-like tau, we used flow cytometry analysis to examine the effect of formaldehyde-aggregated tau on rat hippocampal cells (Fig. 9). Cells were incubated with tau aggregates (after complete removal of formaldehyde traces, see Methods) and then treated with the dyes Annexin V (AV, an indicator of apoptosis) and propidium iodide (PI, an indicator of cell necrosis) [43]. A clear increase was observed in both the AV positive/PI negative (apoptotic) and AV-positive/PI positive (late apoptosis, early necrosis) populations of primary hippocampal cells during the time course of treatment with amyloid-like tau (Fig. 9B–D). We also investigated the efficiency of amyloid-tau produced by treatment with different concentrations of formaldehyde to induce apoptosis. Incubation for 72 h with amyloid-like tau induced by 0.1%, 0.05% or 0.01% formaldehyde each produced a significant decrease on the proportion of apoptotic cells (23%, 22% and 15%, Fig. 9D–F, respectively).

## Discussion

Methanol is an ocular toxicant that causes visual dysfunction often leading to blindness after acute exposure. The physiological and biochemical changes responsible for this toxicity are poorly understood [44]. According to a recent report, humans are uniquely sensitive to the toxicity of methanol, as they have limited capacity to oxidize and detoxify formic acid. Thus, the toxicity of methanol in humans is characterized by formic acidaemia, metabolic acidosis, blindness or serious visual impairment and mild central nervous system depression or even death [1,44]. This view is based on the following two observations: (1) methanol is metabolized to formaldehyde in liver cells and also in neurons [24-27,45], although it is very rapidly converted to formate; (2) SSAO-mediated generation of formaldehyde can induce protein (i.e. β-amyloid) cross-linkage, deposition and subsequently plaque formation in Alzheimer's disease [29-31].

In recent years, however, formaldehyde has been found to be a neurotoxic molecule and to damage the prefrontal cortex of rats including the hippocampus [46,47]. These results demonstrate the formaldehyde-induced neurotoxicity to neurons. Our studies show that formaldehyde induces neuronal tau to aggregate. Here, we show that amyloid-like tau induces apoptosis of SH-SY5Y and hippocampal cells. In fact, chemically, formaldehyde reacts with thiol (our unpublished data) and amino groups instantly, while misfolding of neuronal tau is a subsequent event. This suggests that amyloid-like tau may be involved in methanol toxicosis, particularly the chronic damage to neurons.

The microtubule associated protein tau plays an important role in maintenance of the cytoskeleton. It promotes and maintains assembly of microtubules, which are required for axonal morphogenesis and transport [48]. In recent years, PHFs, formed by misfolding of tau, were found to be the main component of neurofibrillary tangles involved in neurodegeneration, such as in Alzheimer's disease. PHF-tau is not only commonly found in Alzheimer's brain, but is also induced by simple incubation of native tau with some glycosaminoglycans, for instance heparin, in vitro [5,32]. Here, we found that formaldehyde-treated tau forms amyloid-like aggregates, although not necessarily PHFs. Certainly, under the conditions used, self-aggregated tau showed certain differences in structure compared with the aggregates induced by exposure to formaldehyde. (1) Congo Red assays showed that the dye absorbance increased by 16% after incubation with formaldehyde-treated tau, and the absorbance increase was accompanied by a red-shift to 510 nm. Similarly, when formaldehyde-treated tau was added to ThT, a 4-fold increase in the emission intensity and the emission maximum shifted to 482 nm were observed. In contrast, self-aggregated tau induced little change in the spectra of this amyloid-specific dye. (2) Electron microscopy showed that formaldehyde-treated tau had the appearance of granular amyloid-like aggregates, with the diameters in the range of 20–100 nm, unlike fibrillary structures in PHF-tau. (3) The results observed by AFM further confirmed the presence of globular aggregates under the same conditions. The results suggest that formaldehyde promotes the formation of amyloid-like aggregates, which may represent a variant of tau amyloid-like structure.

Recently, Kuret and colleagues described a tau assembly pathway in which anionic inducers, for instance arachidonic acid (AA), favor a shift in the equilibrium between unfolded and filamentous tau species. The microtubule binding function of tau is lost and tau protein accumulates in a partially folded, ThT-positive intermediate which then self-aggregates into a hydrophobic nucleus (as detected by fluorescent ANS), before the filament nucleus elongates to form full fibrils [37,39]. In contrast, formaldehyde-tau was not observed to elongate into filaments on the experimental timescale used in this paper. However, as formaldehyde is not an anionic inducer, it is not surprising that different characteristics are observed between formaldehyde- and AA-induced tau aggregates.

As shown above, formaldehyde reacted with the amino groups of tau, as demonstrated by the OPT test. Reaction with formaldehyde is known to eliminate positive (NH_2_) groups and to increase the net negativity of a protein [49], which may lead to conformational changes in protein tau. Unlike tau, however, formaldehyde at the low concentrations used here did not induce any detectable degree of aggregation or conformation change in BSA. According to Schweers *et al*. (1994) [48], the conformation of native tau features a "worm-like" or a "denatured-like" structure, leaving ε-amino groups of Lys exposed to the exterior of the tau molecule, which would allow formaldehyde to interact with the amino groups of tau. Furthermore, it has been reported that neuronal tau is prone to aggregation when incubated at 37°C or room temperature for over 10 h [5,32]. On the other hand, in BSA, a globular protein, not all of the ε-amino groups are accessible for reaction with formaldehyde. As a crosslinking agent for globular proteins, formaldehyde is not so particularly efficient. Glutaraldehyde is commonly used because the linker region is long enough to bridge two protein molecules. The fact that neuronal tau is prone to aggregate when exposed to low concentrations of formaldehyde, probably reflects the unfolded nature of its native conformation.

Khlistunova and colleagues found that the repeat domains of intracelluar tau could aggregate and were toxic to neuronal cells. The degree of tau aggregation and toxicity depends on the propensity to form β-structure [15,38,50,51]. In the present study, we found that extracelluar tau aggregates can induce neuronal cell apoptosis, similar to the results obtained with extracelluar amyloid or α-synuclein [7,8,43,52,53]. This suggests that structures enriched in β-sheet are important for amyloid-like protein aggregation and neurotoxicity. Hence intracelluar amyloid-like proteins can form neurotoxic aggregates in vitro. In our experiments, a low concentration of formaldehyde induced recombinant tau to aggregate into cytotoxic amyloid-like granular aggregates, providing a new potential mechanism for tauopathies. However, our work provides an effect on protein tau aggregation in vitro of low concentrations of formaldehyde. For an in vivo environment where many other biochemical and biophysical factors exist and interact with each other, further investigation needs to becarried out.

## Conclusion

Here we investigate the effect of low concentrations of formaldehyde on protein misfolding and aggregation. We found that unlike the typical globular protein BSA, the natively-unfolded structure of human neuronal tau was induced to misfold and aggregate in the presence of 0.01% formaldehyde, leading to formation of amyloid-like deposits that appeared as densely staining granules by electron and atomic force microscopy, and bound the amyloid-specific dyes thioflavin T and Congo Red. After removal of the formaldehyde, the amyloid-like aggregates of tau were found to induce apoptosis in the neurotypic SH-SY5Y cells and in rat hippocampal cells, as observed by Hoechst 33258 staining, assay of caspase-3 activity, and flow cytometry using Annexin V and Propidium Iodide staining. Control cells incubated with formaldehyde alone, or with tau aggregates formed in the presence of acetaldehyde or in the absence of additives (and which did not show appreciable binding of thioflavin T or Congo Red), did not show signs of apoptosis. Further experiments showed that Congo Red specifically attenuated the caspase-3 activity induced by amyloid-like deposits of tau. The results suggest that low concentrations of formaldehyde may play a role in induction of tauopathies.

## Methods

### Materials

The clone of recombinant human tau-40 was kindly provided by Dr. Goedert (University of Cambrige, UK) [4]. 1-anilinonaphthalene-8-sulfonic acid (ANS), Thioflavin T (ThT), Congo Red and 3-[4,5-dimethylthiazol-2-yl]-2,5-diphenyl-tetrazolium bromide (MTT) were from Sigma. Sephadex G-50, Q-Sepharose and SP-Sepharose came from Pharmacia. Ultra pure formaldehyde and acetaldehyde were from Acros. Anti-tau monoclonal antibody Tau-1 (MAB3420) came from Chemicon. Dulbecco'd modified Eagle's medium (DMEM) came from NUNC. AC-Asp-Glu-Val-Asp-paranitroaniline (Ac-DEVD-pNA) was from Calbiochem. BSA came from Boehringer. All other reagents were analytical grade and were used without further purification.

### Expression and purification of recombinant htau-40 and microtubule binding assay

Neuronal tau, overexpressed in *E. coli*, was purified as described previously [54-56]. Briefly, the bacterial cells were homogenized with a sonicator, boiled at 100°C and the protein was purified using Sepharose-Q, Sepharose-SP and Sephdex-50 chromatography columns. The concentration of recombinant tau was determined spectrophotometrically by measuring the absorbance at 280 nm [57] and the protein appeared as a single band in SDS-PAGE after purification. Assay of tau was performed with tubulin according to Alonso *et al*. [3]. Tau (4 μM) was mixed at 4°C with purified porcine brain tubulin (1 mg/ml) and 1 mM GTP, all in polymerization buffer (100 mM MES, pH 6.7, 1 mM EGTA, and 1 mM MgCl_2_) in a final volume of 500 μl. Once mixed, the samples were pipetted into quartz microcuvettes (1 ml), equilibrated at 37°C, and the absorbance at 350 nm was measured on a Hitachi U-2010 spectrophotometer. The specific activity of tau was the same as that described by Goedert *et al*. (1990) [58].

### Light scattering and electrophoresis

For protein aggregation experiments, tau (1.2 μM for light scattering or 20 μM for electrophoresis) was incubated at 37°C with different concentrations of formaldehyde in 100 mM phosphate buffer (pH 7.2) for 24 h to allow the reaction to reach completion. Acetaldehyde and BSA were used as controls. For time course analysis, aliquots were taken at different time intervals during incubation of tau with or without formaldehyde. Light scattering was measured in a Hitachi F-4500 fluorescence spectrophotometer (slits: Em = 5.0 nm and Ex = 5.0 nm) with excitation at 480 nm. Kinetic data were analyzed according to Tsou (1965) [59]. Electrophoresis equipment was from Bio-Rad.

### Fluorescence and CD measurements

To test conformational changes, ANS was employed to detect whether the aggregates of formaldehyde-treated tau had exposed hydrophobic surface area. The fluorescence at 480 nm of ANS after excitation at 350 nm for different concentrations of tau was determined at room temperature using a Hitachi F-4500 spectrofluorometer. CD spectra were recorded using a Jasco J-720 CD spectrometer. The spectra were measured in 1-mm pathlength quartz cuvettes, and data were collected from 195 nm to 250 nm at 0.5-nm intervals. The samples were all in 100 mM sodium phosphate buffer (pH 7.2). The bandwidth was set at 1.5 nm (37°C). The spectrum baselines were corrected using the spectrum for the buffer measured under identical conditions.

### OPT modification

Neuronal tau (final concentration 0.1 μM) was resuspended in 50 mM phosphate buffer containing OPT (molar ratio: reagent/protein = 20/1) in the presence of formaldehyde at different concentrations at 37°C for 120 min. The fluorescence (Ex340 nm/Em455 nm) was then measured (slits: Em = 5.0 nm and Ex = 5.0 nm). Under the same conditions, tau was resuspended in 0.005% formaldehyde and 2 μM OPT; and aliquots were taken to measure the fluorescence at different time intervals. The data were analyzed according to Tsou (1965) [59].

### Congo red and thioflavin T binding assays

The assay [36] was performed by adding a freshly prepared stock solution of Congo red in 100 mM potassium phosphate (pH 7.2) to tau samples (2 μM) to give a final Congo Red concentration of 5 μM. The ThT assay was performed by adding a freshly prepared solution of ThT in the phosphate to tau samples to give a final ThT concentration of 10 μM [35]. The protein absorbance contribution was subtracted from the spectra.

### Electron microscopy

For electron microscopy, tau (40 μM) was incubated with 0.1% formaldehyde at 37°C for 24 h. The incubated samples were loaded on a carbon-coated grid for 2 min, stained with 2% (w/v) uranyl acetate for 1 min, and then dehydrated through a graded water-ethanol series. Samples were visualized under a JEOL JEM-100CX electron microscope. Tau alone (40 μM) was incubated under the same conditions in the presence of heparin (1 mg/ml) as a control.

### Atomic force microscopy

Neuronal tau (final concentration 10 μM) was incubated at 37°C with formaldehyde (0.05%) in 25 mM phosphate buffer (pH 7.2) over night. Then protein solution was diluted using phosphate buffer and 3 μl of the sample (10 ng tau protein) was dropped onto the mica surface and left for 5 min at room temperature before drying with nitrogen gas. The mica diaphragm was rinsed with ultra-purified water 20 times and dried down with nitrogen gas before observation under the atomic force microscope (Mutiplemode-I, Digital Instruments). The horizontal diameter at half height of a particle (globular protein) was measured and the data were analyzed using the Nanoscope 6.11r1 software.

### Cell culture

SH-SY5Y human neuroblastoma cells were cultured in DMEM medium supplemented with 100 IU/ml penicillin and 100 μg/ml streptomycin at 37°C in a humidified 5% CO_2 _incubator, as described [60]. The medium contained 10% newborn calf serum. Cells were grown to 70–80% confluence in 25 mm diameter dishes and fed every fourth day. Rat hippocampal cell cultures were established from 18-day embryos, as described previously [43]. Briefly, the hippocampi of the 1-day-postnatal Sprague-Dawley rats were microscopically collected, digested in 0.025% trypsin and mechanically dissociated, and the cells were plated on poly-L-lysine-coated plastic dishes (at a final density of 2 × 10^6 ^cells/ml) or glass coverslips (at a final density of 2.5 × 10^5 ^cells/ml). Cultures were maintained in Dulbecco's modified Eagle's medium (DMEM) supplemented with 10% fetal bovine serum and 10% fetal horse serum. For most experiments, the culture medium was replaced with serum-free medium before the addition of formaldehyde-treated tau. Native tau and acetaldehyde-treated tau were used as controls. Cells were incubated with the samples for 72 h. As an additional control, cells were treated with an equivalent amount of 100 mM phosphate butter (pH 7.2) in the absence of tau, and no effect was detected. To inhibit apoptosis, Congo Red was incubated with the cells for 5–10 min before adding the protein of interest. Photos were taken using an Olympus IX-71 inverted contrast microscope.

### Cell viability detection

As described by Mayo *et al*. [41], viability was assessed by reduction of MTT. MTT is a water-soluble tetrazolium salt, reduced by metabolically viable cells to a colored, water-insobule formazan salt. Cells were grown in 96-well plates and treated with different concentrations of aldehyde-aggregated tau or formaldehyde as described above, with eight wells per experimental condition. The concentration of aldehyde-aggregated tau used was 2 μM according to the report that endogenous tau is present at 8–12 μM in human brain [61]. After adding MTT (0.5 mg/ml final concentration) to the culture medium of the cells, plates were incubated at 37°C for 30 min. The assay was stopped by replacement of the MTT-containing medium with 100 μl dimethysulfoxide (DMSO). Absorbance at 595 nm was read by means of an ELISA plate reader. Each experiment was repeated at least three times.

### Apoptosis detected by Hoechst 33258 staining and assay of caspase-3 activity

After incubation with tau, the formaldehyde was removed from the protein sample by a series of dilution steps in an Amicon Microcon-10 column. In order to verify the absence of formaldehyde before use, the presence of the aldehyde group was detected as described [62], and no residual formaldehyde was detected in the sample. Cells were collected by centrifugation and the pellets were washed twice with PBS. Pellets were then re-suspended in PBS and stained with 10 μg/ml Hoechst 33258 for 10 min at room temperature. Morphological evaluation of nuclear condensation and fragmentation was performed immediately after staining with a Nikon Microphot-FXA fluorescence microscope. Colorimetric assay of caspase-3 was performed using a kit from Clontech, as described previously [63]. Briefly, aliquots of cytosolic extracts (20 μg protein in 100 μl caspase-3 assay buffer consisting of 50 mM Hepes, pH 7.4, 100 mM NaCl, 0.1% CHAPS, 10 mM DTT, 1 mM EDTA, and 10% glycerol) were mixed with equal volumes of 40 μM colorimetric tetrapeptide substrate (Ac-DEVD-pNA) in the same buffer and monitored using an ELISA plate reader.

### Flow cytometric analysis

Cells undergoing apoptosis were detected with the use of double staining with Annexin V-FITC/PI in dark according to the manufacturer's instructions [43]. Briefly, cells attached to plastic dishes were harvested by 0.25% trypsin and washed twice with cold PBS. The cell pellets were suspended in 1× binding buffer (10 mM HEPES/NaOH, pH 7.4, 140 mM NaCl, 2.5 mM CaCl_2_) at a concentration of 1 × 10^6 ^cells/ml. Then the cells were incubated with AnnexinV- FITC and propidium iodide (PI) for 15 min (22–25°C) in dark. The stained cells were immediately analyzed by flow cytometry (FAC Svantage SE, USA). Annexin V-FITC selectively passed through the plasma membranes of apoptotic cells and stained them with green fluorescence. Apoptosis was considered to have taken place in cells positive for Annexin V-FITC and negative for PI. All data were analyzed with Cell Quest software (BD). Each measurement was carried out at least in triplicate.

## Authors' contributions

CLN was responsible for the experiments, data analysis and drafted the manuscript. XSW conducted the electron microscopy assay, and contributed throughout the experimental process. YL and SP drafted portions of the text. RQH participated as a supervisor in the study design, analyses and writing. All authors read and approved the final manuscript.

## Figures and Tables

**Table 1:** Activity of tau aggregates in tubulin assembly

Tau protein	Specific Activity (A_350_/s· mg × 10^-3^)	Relative Activity (%)
Native tau	40.0 ± 1.02	100
Acetaldehyde tau	30.0 ± 1.98	75
Self-aggregated tau	10.0 ± 2.10	25
Formaldehyde tau	2.0 ± 0.28	5
Tubulin	1.3 ± 0.21	3

Specific activity was quantified by absorbance at 350 nm and the results represent the means ± S.D. Relative activity was as a percentage.
